# Bioaccumulation of Organic and Inorganic Pollutants in Fish from Thermaikos Gulf: Preliminary Human Health Risk Assessment Assisted by a Computational Approach

**DOI:** 10.3390/jox14020041

**Published:** 2024-06-01

**Authors:** Konstantinos M. Kasiotis, Effrosyni Zafeiraki, Electra Manea-Karga, Demetrios Kouretas, Fotis Tekos, Zoi Skaperda, Nikolaos Doumpas, Kyriaki Machera

**Affiliations:** 1 Laboratory of Pesticides’ Toxicology, Department of Pesticides Control and Phytopharmacy, Benaki Phytopathological Institute, 8 Stefanou Delta Str., 14561 Athens, Greece; e.manea-karga@bpi.gr (E.M.-K.); k.machera@bpi.gr (K.M.); 2 Department of Biochemistry and Biotechnology, University of Thessaly, Viopolis, Mezourlo, 41500 Larissa, Greece; dkouret@uth.gr (D.K.); fotis.tek@gmail.com (F.T.); skaperda@foodoxys.com (Z.S.); 3 iSea, Environmental Organisation for the Preservation of the Aquatic Ecosystems, 54645 Thessaloniki, Greece; nikolaos.doumpas@isea.com.gr

**Keywords:** fish, pesticides, pharmaceuticals, heavy metals, Mediterranean, liquid chromatography, gas chromatography, inductive coupled plasma, mass spectrometry

## Abstract

The monitoring of contaminants in fish species is pivotal for fishes’ health and reproduction, as well as for human health. In the specific work, three major categories of contaminants, pesticides, pharmaceuticals, and macro and trace elements, were investigated in two major fish species, *Dicentrarchus labrax* and *Solea solea*, collected from Thermaikos Gulf, in Greece. To achieve this goal, three analytical methods using LC-MS/MS, GC-MS/MS, and ICP-MS were developed, validated, and applied to the collected fish samples. The results indicated a very low prevalence of caffeine and acetaminophen, both not exceeding 3.8 μg/kg fish. Similarly, thiabendazole, cypermethrin, and tricyclazole (pesticides) were found in a concentration range of 0.9 to 13.7 μg/kg fish, while in one *D. labrax* sample, traces of the metabolite of organochlorine pesticide DDT, *o,p’*-DDE were detected. Al, Mn, Fe, Zn, and Sr were the predominant trace elements in a concentration range of 500–20,000 μg/kg fish. Macro elements levels varied from 280 to 5405 mg/kg fish. Health risk assessment did not unveil an unacceptable risk for the human health of adults, apart from one sample presenting Hg above the regulatory levels. On the contrary, for children, the calculated hazard quotient values for Hg in all cases and for two As detections were higher than the threshold value of 1, indicating a potential risk.

## 1. Introduction

Marine pollution has gained scientific interest over the last decades, as the presence of pollutants agitates the natural balance of the ecosystems and may pose a threat to the environment and to human health. Coastal areas, including estuarine, bays, and gulfs, are considered to be more vulnerable to the accumulation of pollutants, since they are heavily impacted by both natural and anthropogenic activities [1]. The European Union’s Marine Strategy Framework Directive (MSFD) has set a clear framework for the maintenance of proper marine environmental status, embracing quality descriptors such as biodiversity status and contaminant levels in fish [2]. In this context, the investigation of chemicals (such as pharmaceuticals) in the marine environment has long been an objective of many studies and was reviewed recently [3], with compounds such as acetaminophen and ibuprofen holding a main position among the analgesic compounds and non-steroidal anti-inflammatory drugs, respectively, frequently found in seawater, including the Mediterranean Sea, with relatively high concentrations [4,5]. Similarly, heavy metals and pesticides are also at the forefront, with the described undesired health effects on fish commodities and aquatic organisms in broader sense [6,7,8,9,10] included in marine monitoring schemes [11,12,13].

Thermaikos Gulf is a semi-closed shallow gulf in the northwestern Aegean Sea, characterized by intense activities taking place in its coastal area. Thermaikos Gulf is the recipient of the input of four rivers (Axios, Aliakmon, Gallikos and Loudias) and two municipal wastewater treatment plants of Thessaloniki. Additional pressure received by the discharges of industrial, agricultural, marine, and aquaculture activities occurring at the coastline of the gulf, makes the latter a reservoir of pollutants. The elevated chemical burden in the marine and coastal environment of the Gulf yields effects on both marine ecosystem and humans [14]. Since fishing and mussel farming are economic drivers for the coastal cities of Thermaikos (from Thessaloniki to Larissa) [15], the required balance between such activities and ecological and socioeconomic targets should not be disregarded, and is usually safeguarded by marine spatial plans [16]. Considering that Thermaikos Gulf is one of the major fishing grounds of Greece and this part of Mediterranean [14,17,18,19], the occurrence of potentially toxic chemicals in aquatic organisms can provoke great health risks to the consumers.

According to the literature, many undesirable substances, such as pesticides, pharmaceuticals, and heavy metals, are absorbed and finally accumulate in the marine organisms [20,21]. Due to the toxicity of these pollutants, their presence in fish is of critical significance. However, the available information on organic and inorganic pollutants in fish from Thermaikos Gulf is still limited, with most of the studies focusing on this area providing data related to the sediment, algae, and water pollution [22,23,24,25,26,27].

Identifying the need for control strategies and continuous monitoring of pollutants in aquatic organisms in Thermaikos Gulf, in the present study, we elaborate the detection of several organic and inorganic pollutants in the euraline fish species, *Dicentrarchus labrax* (*D. labrax*, European seabass) and *Solea solea* (*S. solea*, common sole). In particular, 286 chemicals, including pesticides, pharmaceuticals, trace, and macro elements, were incorporated into the analytical methods to investigate the occurrence of the former and further evaluate the impact of the pressure upon the Thermaikos ecosystem. The potential risk to human health posed by the most hazardous compounds or elements detected was also assessed and discussed. Overall, the herein concurrent application of several multi-analyte methods covering a broad range of chemicals in marine organisms is of high significance due to the global need for the generation of contaminants data that, to an extent, portray the contaminants’ burden on the sampled area and in parallel feed health risk assessments.

## 2. Materials and Methods

### 2.1. Samples

**Figure 1:**
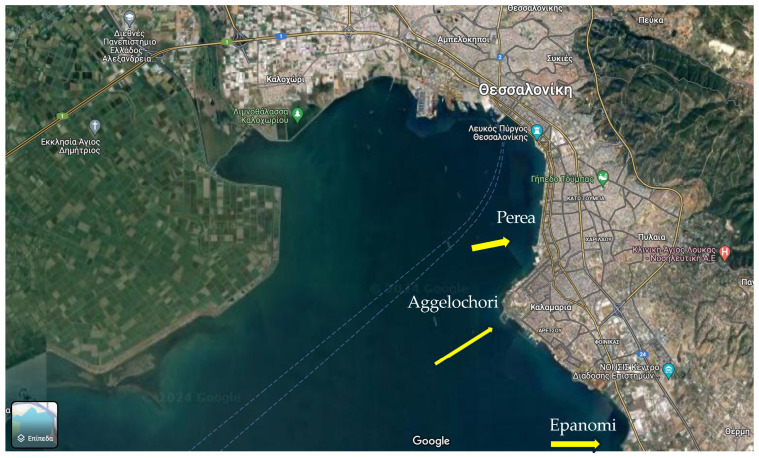
Map of sampling points (photo retrieved from Google Maps).

Fish samples of *D. labrax* and *S. solea* [28] were collected from the internal part of Thermaikos Gulf (in proximity to the coastal line of Thessaloniki, see Figure 1) in the areas of Perea, Aggelochori, and Epanomi during spring 2021 following a well-established protocol [29] and were placed into distinct resealable bags with a label declaring pertinent information (e.g., date, time, site, species). Since samples treatment/dissection was not possible within 24 h, samples were frozen at −20 °C. Prior to chemical analysis, samples were unfrozen, and the whole fish was homogenized.

### 2.2. Chemicals, Materials, and Standard Solutions

Some of the chemicals used for the analysis of pesticide residues were previously reported in other published works from our group [30,31,32,33]. Enhanced matrix removal (EMR)-lipid clean-up dispersive SPE (dSPE) was purchased from Agilent Technologies. The mass-labeled internal standards, carbendazim D3, imidacloprid-D4, dimethoate-D6, and chlropyrifos-D10 were used for the quantification of the compounds measured with LC-ESI-MS/MS, and they were purchased from Sigma Aldrich (Seelze, Germany). Triphenyl phosphate (TPP) and dichlorvos-D6 were used in the GC-MS/MS analysis, and were acquired from Sigma-Aldrich (Seelze, Germany). The overall analytical scope covered approximately 250 pesticides (see Table S1 for the analytes included). In total, 4 Antibiotics and 6 pharmaceuticals were selected to be monitored, taking into consideration the prevalence of such compounds in wastewater and the marine environment in general, including the Mediterranean Sea [4,11,34]. Chemicals included were acetaminophen, amoxicillin, azithromycin, atorvastatin, caffeine, cefuroxime, clarithromycin, ibuprofen, ramipril, and venlafaxine. All analytical standards were obtained from Sigma Aldrich (Seelze, Germany) and were of a purity >99%. Distinct stock solutions at 1000 μg/mL were prepared in organic solvent (MeOH for the LC-ESI-MS/MS, and acetone or acetonitrile for GC-MS/MS), and intermediate/working solutions were prepared by appropriate dilutions in respective solvents.

Twenty seven (27) elements were determined and quantified by using a standard mix of 25 chemical elements (Al, Ba, B, Cu, Fe, Sr, Zn, Be, Cr, Co, Li, Mn, Mo, Ni, Ti, V, Sb, As, Cd, Pb, Se, Ag, Tl, Sn, and U in HNO_3_ 5%) and standards solutions of individual elements (Hg in 10% HNO_3_, P in 0,05% H_2_SO_4,_ Mg, and Ca in 2% HNO_3_), purchased from CPAchem (Stara Zagora, Bulgaria). Internal standards: individual solutions of Lithium (6Li), Scandium (Sc), Germanium (Ge), Ytrium (Y), Indium (In), Terbium (Tb), and Iridium (Ir), and a standard solution/mixture of 25 element components (Al, Be, Co, Li, Se, Sn, Zn, Sb, B, Cu, Mn, Ag, Ti, As, Cd, Fe, Mo, Sr, U, Ba, Cr, Pb, Ni, Tl, and V in HNO_3_ 5%) were used for the preparation of quality assurance (QC) standard and were also provided by CPAchem (Stara Zagora, Bulgaria). The European Reference Material, ERM^®^-BB422 fish muscle, was obtained from the Joint Research Centre (JRC) of the European Commission (Certified Reference Material (CRM), fish muscle fortified with 14 elements at known concentration, JRC, Institute for Reference Materials and Measurements, Geel, Belgium). Speciation analysis for As and Cr (both organic and inorganic forms) was not performed during this study. Nitric acid (HNO_3_) 67–69% w/w and hydrogen peroxide (H_2_O_2_) 30% w/w (trace metal grade) were all purchased from Seastar Chemicals Inc. (Sidney, Canada). Ultrapure water from an ultrapure Milli-Q water system (Burlington, MA, USA) was also used for the dilution of all the solutions.

### 2.3. Sample Preparation

#### 2.3.1. Pesticide Residues and Pharmaceuticals Analysis

Blank fish samples of both fish types (of organic fish farming) were obtained from the market, since, to our knowledge, a CRM-negative fish tissue was not available (taking into account all classes of chemicals screened in this work). The samples were homogenized in their totality and used to validate the analytical methods. Prior to the validation of the methods, the samples underwent sample preparation to verify the absence of the chemicals studied herein. Since no significant difference in terms of matrix interferences between the two types of fish was obtained, only one was used for thorough validation trials.

The sample preparation for the determination and quantification of pesticides and pharmaceuticals was based on a previous work from our team [30], applying some modifications proposed in the literature concerning the more efficient removal of lipids in lipid-rich matrices [35,36]. More specifically, homogenized fish (2 g whole fish) were placed inside a falcon tube (50 mL) and spiked with six internal standards, carbendazim-D3, imidachloprid-D4, dimethoate-D6, dichlorvos-D6, chlorpyriphos-D10, and TPP at a concentration of 50 ng/g. Then, ACN (10 mL), MgSO_4_ (2 g), and 1 g sodium acetate were introduced sequentially, and the tube was vigorously hand-shaken for 1 min and vortex-mixed for 3 min to achieve homogenization. Consequently, the mixture was centrifuged for 10 min at 4500 rpm (Hettich Zentrifugen, UNIVERSAL 32R, Tuttlingen, Germany) with a stable temperature of 10 °C. The upper layer was transferred to another falcon tube (15 mL), where EMR lipid agent (500 mg) and 3 mL H_2_O were placed. The mixture was vortex-mixed for 1.5 min and centrifuged for 5 min at 4500 rpm and 10 °C. The supernatant was divided into two parts, and both aliquots were evaporated to dryness using a N_2_ stream at 35 °C. Consequently, the dried extracts were reconstituted with 1 mL of a 75:25 (*v*/*v*) MeOH:H_2_O and 1 mL of pure acetone and injected into the LC-MS/MS and GC-MS/MS system, respectively (before injection filtering through a PTFE filter (0.22 μm) was performed). Considering the acceptable recoveries obtained during method validation and to reduce the analysis cost, additional internal standards of the pharmaceuticals were not acquired.

#### 2.3.2. Trace and Macro Elements Analysis

For the analysis of trace and macro elements in fish samples, 0.5 g of every homogenized fish sample was weighed and placed into a microwave oven vessel (ETHOS UP, Milestone) for digestion. HNO_3_ (9 mL) and H_2_O_2_ (1 mL) were added in each vessel, and the samples were further digested under 210 °C and 1800 W for 35 min. The vessels were then cooled to less than 40 °C, and the digestion solution was transferred to a clean container. Ultrapure water was added until a final volume of 100 mL. All the labware and the vessels were acid-washed (immersion into a diluted HNO_3_ 0.05% with ultrapure water) for 24 h and then washed several times with ultrapure water. Then, they were dried in a clean hood at room temperature.

### 2.4. Instrumental Analysis

#### 2.4.1. Liquid Chromatography Electron Spray Ionization Tandem Mass Spectrometry (LC-ESI-MS/MS)

An Agilent triple quadrupole (6410B, QQQ) system was used for the analysis of the samples and the detection of pesticide residues. The LC was equipped with a C18 column, and the chromatographic gradient started from 80% mobile phase A (% 5 mM ammonium formate in H_2_O, 0.1% formic acid) to 100% mobile phase B (5 mM ammonium formate in MeOH) in 30 min. An injection volume of 20 µL was applied, while the flow rate was set at 0.28 mL/min. Results were also corroborated in an additional triple quadrupole system, namely a triple Quad LC/MS system (Shimadzu LCMS-8060 NX, Kyoto, Japan), using the same conditions described above, considering the enhanced performance of this system in terms of sensitivity for some of the analytes (i.e., tricyclazole and ibuprofen).

#### 2.4.2. Gas Chromatography Tandem Mass Spectrometry (GC-MS/MS)

The GC-MS/MS analysis of the samples was performed on a Shimadzu triple quadrupole mass spectrometer GCMS-TQ8040NX composed of a GC-2030 gas chromatograph. The GC system was equipped with an autosampler AOC-6000 (CTC Analytics) and a 10 μL syringe. The capillary column used for the chromatographic separation was the MEGA-5 HT (30 m × 0.25 mm × 0.25 μm) (MEGA S.r.l., Legnano, Italy), and the chromatographic gradient applied was the following: initial oven temperature of 50 °C for 1 min, increase in T until 125 °C at a rate of 25 °C/min, and finally reach of 300 °C at a rate of 10 °C/min. The analyses in MS/MS were performed in multiple reaction monitoring (MRM) mode.

#### 2.4.3. Inductive Coupled Plasma Mass Spectrometry (ICP-MS)

The ICP-MS analysis was performed on a Thermo iCAP-RQ equipped with an ASX-280 autosampler, as previously described in detail [37]. Briefly, the instrument used a Ni sample and skimmer cones, a Quartz cyclonic spray chamber, and a MicroMist U-Series Nebulizer (0.4 mL/min with PEEK connector). The flow of the nebulizer gas and the cool gas was approximately 1 L/min (autotune dependent) and 14 L/min, respectively. Prior to the analysis, the system was equilibrated, and a performance report in KED mode was performed. In this context, the analysis was conducted with high sensitivity and stability, while the occurrence of interferences and doubly charged ions was minimized.

### 2.5. Quantification and Quality Assurance

#### 2.5.1. Pesticide Residues

The two multiresidue methods of LC-ESI-MS/MS and GC-MS/MS were validated based on the SANTE Guidance Document on analytical quality control and method validation procedures for pesticide residues analysis in food and feed [38]. The method limit of quantification (LOQ) for pesticides was established as the lowest fortification level that provided sufficient accuracy and precision. The LOQ and recovery values for each active substance are presented in Table S1. Three control samples were analyzed in every sequence of samples, and no pesticide residues or their metabolites were detected in any of them. All validation metrics, such as selectivity, working concentration range and linearity, LOQ, precision, and accuracy, were determined following the steps described in the SANTE guidance document.

#### 2.5.2. Pharmaceuticals

For pharmaceuticals, method validation was also based on the SANTE document [38]. Yet, the limits of detection LODs and LOQs were estimated from the analyte signals observed after LC-ESI-MS/MS analysis of fortified fish samples, as the concentration that provided a signal-to-noise ratio of 3 in the case of the LOD and 10 in the case of the LOQ (presented in Table S2). The linearity was evaluated through seven calibration points, namely 1, 5, 10, 20, 50, 100, and 200 ng/mL, and considered acceptable when the regression coefficient (R^2^) was above 0.99. MRM conditions, including collision energies applied and RT for each pharmaceutical, are illustrated in Table S3. For both pharmaceuticals and pesticides, quantification was conducted with external standard calibration using matrix-matched calibration curves.

#### 2.5.3. Trace and Macro Elements

Solvent calibration curves covering concentrations from 0.1 to 1000 μg/Kg and from 0.1 to 200 mg/Kg were prepared for the quantification of trace elements (Al, Ti, V, Cr, Mn, Fe, Co, Ni, Cu, Zn, As, Se, Sr, Mo, Ag, Cd, Sn, Sb, Ba, Tl, Pb, Hg, and U) and macro elements (Mg, P, K, and Ca), respectively. The regression coefficient (r^2^) was greater than 0.995 for all the calibration curves, while seven internal standards (^6^Li, Sc, Ge, Y, In, Tb, and Ir) were added to all unknown samples and calibration standards at a constant rate. The limits of quantification (LOQ) values and the quantification parameters for each element are presented in Table S4. Two quality control (QC) standards solutions of macro and trace elements were analyzed in every batch of samples, monitoring the repeatability of the analytical method. The recovery of the two QC standards ranged between 80–120% in all the samples. In addition, blank reagents, spiked samples, and ERM were analyzed following the same procedure as the unknown samples and measured in every sequence to control the background contamination and the accuracy of the method. Moreover, one additional isotope was measured when possible to avoid spectral interferences. To compare the metal concentrations between the two fish species, a one-way analysis of variance (ANOVA) was conducted. The two fish species were set up as the independent variable and the metal concentration was set up as the dependent variable. This allowed us to evaluate whether there were significant differences in metal concentrations between the two species.

### 2.6. Health Risk Assessment

The risk on human health posed by each compound and element detected in the analyzed fish samples was assessed based on the Hazard Quotient (HQ) and Hazard Index (HI) approach [39]. For the calculation of the HQ, the following equations were applied:

(1) $$\mathrm{HQ}=\frac{\mathrm{ADD}}{\mathrm{TDI}}$$

(2) $$\mathrm{ADD}=C\times \frac{\mathrm{IR}}{\mathrm{BW}}$$

where:

ADD: average daily intake (μg/Kg bw/d).

TDI: the tolerable daily intake (μg/Kg bw/d) set by European Food Safety Authority (EFSA) and open literature.

C: the mean of each compound or element concentration detected in fish (μg/Kg).

IR: the daily fish consumption rate (Kg/person/d) for Greece (fish: 0.056 Kg/inhabitant/day, [40]).

BW: the mean body weight (70 Kg for adults and 15 Kg for children).

For the estimation of the cumulative health risk, the Hazard Index approach (HI, unitless) was applied (Equation (3)). More specifically, the hypothesis of dose additivity was calculated by summarizing the individual HQ values.

(3) HI=∑HQs=HQ1+HQ2+HQ3+⋯+HQn

### 2.7. In Silico Calculations

Nexus software (version 2.5.1, Lhasa Limited) and Derek Nexus software (version 6.2.0, Lhasa Limited) were used for the in silico approach to corroborate the findings on pharmaceuticals and their relation to health risk assessment. More specifically, more than 60 endpoints were selected, including carcinogenicity, cardiotoxicity, developmental toxicity, hepatotoxicity, neurotoxicity, and teratogenicity. Concerning species, both mammals and bacterium were regarded. For each compound, structural details exemplified by LogP, LogKp, exact, and average molecular mass were considered, while tautomers and mixtures (for the same compound) were also deemed in computations. The Derek KB 2022 1.0 knowledge base was used to retrieve toxicological information.

## 3. Results

### 3.1. Analytical Methods Validation

The fortification experiments resulted in acceptable recoveries for all analytes in the range of 64–117% (see Table S1 for pesticides and Table S2 for pharmaceuticals). Linearity was acceptable with regression coefficients (R^2^) values >0.99 (with the exception of paclobutazole and diphenylamine, where R^2^ were 0.9889 and 0.9881, respectively) accompanied by acceptable residual values. Since a CRM-negative fish tissue sample was not obtained (see Section 2.3.1), focus was given to the assessment of blanks used in this study. The blank extracts were injected into the analytical instruments, and the analytical signal of all analytes was assessed. In limited cases (carbendazim, cyfluthrin, hexaconazole, fenazaquin, oxamyl, mepanipyrim, malathion, fenthion, pymethrozine, pyrazophos, and ramipril), detection of analytes in blank samples was in the range of <LOD to LOQ/4. Therefore, the subtraction of analyte concentration in blank extracts was not deemed necessary. Lastly, the measured concentrations of elements in ERM^®^-BB422 fish muscle were in agreement with the assigned values.

### 3.2. Levels of Pesticide Residues in Fish Samples from Thermaikos Gulf

**Figure 2:**
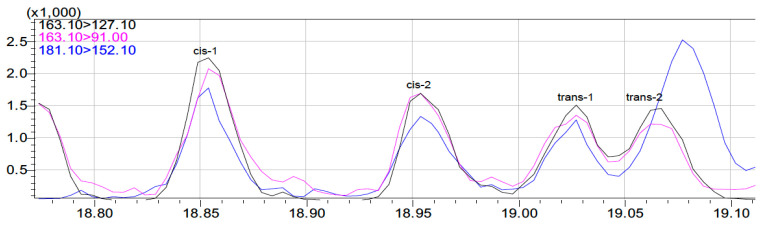
MRM chromatograms of cypermethrin (as a sum of cis and trans diastereomers) in a positive sample.

Eight out of ten samples were devoid of pesticide residues. Surprisingly, in one *S. solea* sample, thiabendazole was quantified at 13.7 μg/Kg fish, which is considered a relatively high concentration to be detected in fish collected from the open sea. Thiabendazole has been reported in South African freshwater impoundment [41], although in the same study, thiabendazole residues were not obtained in fish tissues. Similarly, in Spain, thiabendazole was reported as being among the compounds with the highest concentrations in river water, although not among the most frequently detected [42]. Another azole-type molecule, tricyclazole, was detected in the same sample, but at a lower concentration (at 0.9 μg/Kg fish). On the other hand, cypermethrin was quantified in one sample of *D. labrax* at 8.7 μg/Kg fish (see Figure 2). To our knowledge, for the abovementioned compounds, no MRLs are established in fish commodities by the European Union [43]. Expanding the comparison of findings with the Codex Alimentarius International Food Standard, US-EPA tolerances, and Australian MRLs, similar conclusions were derived, since respective limits in fish are also not established [44,45,46]. Traces of o,p’-DDE (in proximity to the LOD of the method at 0.15 μg/Kg) were detected in one *D. labrax* sample (see Figure S1c).

### 3.3. Levels of Pharmaceuticals in Fish Samples from Thermaikos Gulf

**Figure 3:**
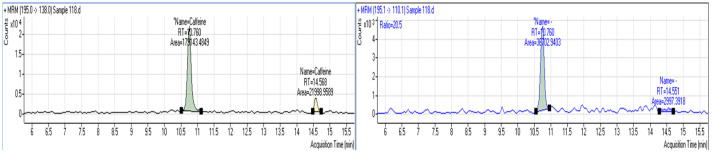
MRM chromatograms of caffeine in a positive sample.

Low prevalence of the selected pharmaceuticals was obtained in the specific work. More specifically, the most prevalent pharmaceutical was caffeine, detected in three samples (two *D. labrax* samples and one *S. solea* sample; for indicative chromatogram, see Figure 3, while for blank and spiked samples, see Figure S2a and b, respectively) in the range of 2.3–3.8 μg/Kg fish. In the same context, acetaminophen was quantified in one sample (*S. solea*) at 1.5 μg/Kg fish. Concomitant detections of pharmaceuticals and pesticides occurred only in one sample (cypermethrin and caffeine in the *D. labrax* sample).

For both caffeine and acetaminophen, no MRL was established in foodstuffs of animal origin. Traces of ibuprofen (at the LOD) were detected in one *D. labrax* sample (see Figure S2c).

### 3.4. Levels of Trace and Macro Elements in Fish Samples from Thermaikos Gulf

**Figure 4:**
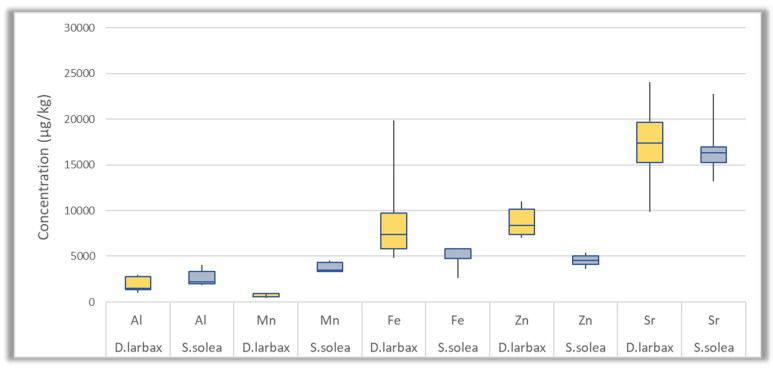
Box plots of the most abundant trace elements (Al, Mn, Fe, Zn, Sr) concentration (μg/Kg) in *D. larbax* (*n* = 5) and *S. solea* (*n* = 5) fish samples.

**Figure 5:**
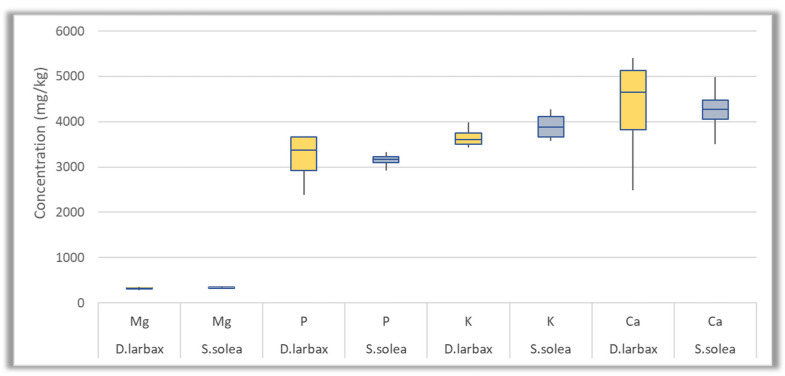
Box plots of macro elements concentration (mg/Kg) in *D. larbax* (*n* = 5) and *S. solea* (*n* = 5) fish samples.

The accumulation of 27 trace and macro elements was investigated in two different species of fish collected from Thermaikos Gulf. The detected concentrations for each individual element are presented in Tables S4 and S5, while the skewness of the most abundant trace elements and of macro elements for each species is also illustrated in Figure 4 and Figure 5, respectively, by displaying the data averages and quartiles. According to the results, trace and macro elements were detected in all the fish samples (*n* = 10). The highest detected concentrations were those of macro elements (Mg, Ca, P, and K), followed by the trace elements Sr, Fe, Zn, Al, and Mn. In particular, the average concentrations of macro elements ranged between 324 and 40,670 mg/Kg fish, while the average values found for the most predominant trace elements in terms of concentration ranged from 2196 to 17,058 μg/Kg fish. On the other hand, the levels of Cd, Sb, U, and Tl were below the LOQ. The rest of the elements showed intermediate levels compared to the aforementioned ones.

**Figure 6:**
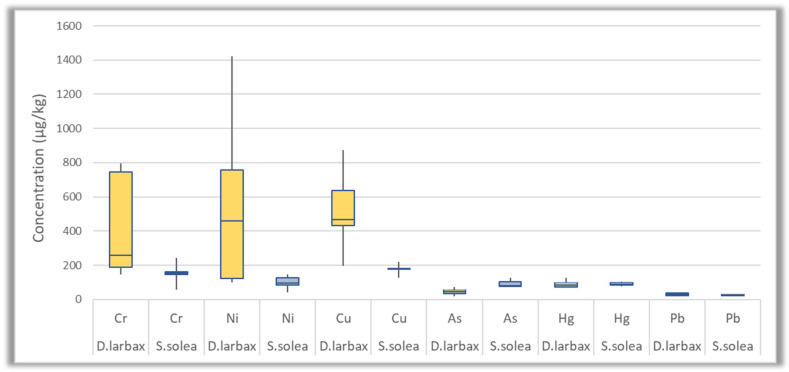
Βox plots of toxic elements concentration (μg/Kg) in *D. larbax* (*n* = 5) and *S. solea* (*n* = 5) fish samples. Cd concentrations are not included, since they were all below LOQ value.

The trace elements, characterized by high toxicity (Pb, Hg, Cd, and total As) were all detected in low concentrations or were not detected at all (concentrations ranged between <LOQ to 128 μg/Kg) in both fish species (Figure 6). This was also the case for the elements that can be both essential and toxic, depending on their oxidation state and concentration (Ni, Cr, and Cu). More specifically, regarding the three latter elements, their detected values were below 873 μg/Kg in all the analyzed samples, except for one sample (sample code: 1, *D. larbax*) that was found to be more contaminated for Ni (Figure 6). With regard to the established maximum levels of Pb (300 μg/Kg fish), Hg (500 μg/Kg fish), and Cd (50 μg/Kg fish) in fish [47], no exceedance was observed in the analyzed samples, although the LOQs for Pb, Hg, and Cd were below the MRLs (see Table S6).

By comparing the contamination levels between the two fish species through the application of an ANOVA test (*p* < 0.05), it was observed that the detected concentrations were of comparable range for all the analytes, except for Cu (*p* = 0.015846), Mn (*p* = 3.23 × 10^−6^), As (*p* = 9.85 × 10^−3^, and Zn (*p* = 9.92 × 10^−4^). In particular, *S. solea* samples were found to have higher concentrations of Mn, while the detected values of Zn and Cu were elevated in *D. larbax*. More specifically, the average concentrations of Mn in *S. solea* were equal to 3785 μg/Kg fish, while in *D. larbax* were 607 μg/Kg fish. The average concentration of As in *S. solea* fish (92 μg/Kg) was approximately twice as high as the concentration detected in *D. larbax* species (46 μg/Kg fish). The opposite was the case for Zn and Cu (average concentrations: 8780 and 522 μg/Kg fish, versus 4538 and 176 μg/Kg fish in *D. larbax* and *S. soela*, respectively). However, the limited number (*n* = 10) of fish samples did not allow us to draw a solid conclusion, and thus further investigation is needed. *S. solea* samples showed higher concentrations for the elements Al, Mn, Fe, Zn, and Sr (range of mean concentrations between 2.7 and 17 mg/Kg), compared to the rest of the trace analytes (mean concentrations below 1 mg/Kg). In another study examining minerals in the tissues of *S. solea* collected from Thermaikos Gulf, it was found that the highest detected levels of elements were observed in the gills, the liver, and the scales of the samples, while the lowest was in their muscle [20]. Since the current fish samples were homogenized, and their tissues were not separated, a direct comparison with the results presented by Vetsis and co-workers [20], was not possible.

### 3.5. Human Health Risk Assessment

Health risk assessment associated with fish consumption and the occurrence of toxic elements, pesticides, and pharmaceuticals residues was estimated, and the values of ADD and HQ are presented in Table 1. To calculate the latter, the TDI (µg/kg/d) values used for all chemicals were retrieved in principle from EFSA and the open literature.

For pesticides and pharmaceuticals, concentrations (μg/Kg fish) were converted to dry weight by multiplying by a factor of 5 [57]. More specifically, the reported HQ values were computed based on the uppermost detected concentrations (worst-case scenario) of the toxic elements and compounds quantified in the analyzed samples. In the same context, the admission of regarding non-edible tissues’, exemplified by the gastrointestinal tract (a reservoir of chemicals [58,59]), contribution to the detected concentrations was made (the sampled fishes were not gutted). Taking these into consideration, in almost all cases, calculated HQ values of elements for adults were below the threshold value of 1, indicating that the consumption of fish does not pose any risk to adults. The only exception accounted for one sample for Hg (HQ = 1.02). On the contrary, when children were considered, HQ values for Hg exceeded the threshold level of 1, which signifies a potential threat. For As in two cases out of ten, HQ values for total As in samples 7 and 8 were found to exceed the threshold level (Table S6).

## 4. Discussion

In view of the above results, differences in concentrations of macro and trace elements and their patterns have been reported in the literature between benthic, demersal, and pelagic fishes [60]. Therefore, the etiology of differences in Zn and Mn levels between the two species can also be attributed to the distinction between *S. solea* (benthic as juvenile and adult) and *D. labrax* (manifests demersal behavior but is more common in shallow waters [61]) and the difference of marine habitat in association to the depth. In a work conducted in the Galapagos marine reserve [60], higher levels of Zn were recorded in pelagic species compared to demersal, which, considering the species collected in this study and their habitat, is in line with the presented findings. In the same context, concentrations of heavy metals were reported to be higher in benthic species compared to pelagic ones [62], possibly due to the proximity of such species to the seabed and the subsequent release and absorption of metals from the sediment (heavy metals usually display higher concentrations at sea bottom compared to the surface water [63]). The latter is in agreement with the presented results for As, in which both the individual and average concentrations in *S. solea* samples were higher than the respective ones in *D. labrax*. For Pb, such a trend was not observed, while for Cd, all values were <LOQ. Nevertheless, based on the bibliography [64], observations should be viewed and deciphered with caution, since the bioavailability of elements is influenced by ecological processes related to specific regions and seasons that can reverse the expected bioaccumulation potential of elements in fishes.

At this point, it is essential to mention that the random and limited sampling (*n* = 10) of the fishes investigated in this study should not be used to draw generic conclusions for the sampled area and fishes and the potential threat to human health. In the same context, Hg (and methyl mercury, CH_3_Hg) is a well-debated topic in the frame of risks and benefits of fish consumption at the forefront of pertinent authorities such as EFSA [65]. As is an element of toxicological concern, with several inorganic forms that are highly soluble in water exhibiting substantial mobility [66], and with numerous sources [67] such as geothermal volcanic activities [68,69], metal mining [70], and other arsenic-generated waste [71]. For Greece in particular, in the islands of the Aegean Sea, there is still active mining and volcanic activity, which, along with the mobility of seawater and capacity to transfer chemicals in large distances, can justify the occurrence and subsequent accumulation of As and other metals in fish tissues [69,72]. In the same context, the topical release of contaminants by ships due to the unintentional release of waste (that contains As and other pollutants) during transportation is also reported, and can be another source of pollution. Nevertheless, the number of analyzed samples is limited, and thus, a more extended study is recommended for a more reliable outcome of risk assessment to be drawn and to better portray the residual prevalence of the sampled area. From the health risk assessment perspective, the inclusion of non-edible tissues in the sample preparation might have impacted the reported concentrations (and findings), especially considering the gastrointestinal tract content known to accumulate chemicals. Nevertheless, this source of uncertainty seems to be “considered” when viewed in the context of a worst-case scenario and the likelihood of slightly elevated concentrations’ insertion in risk assessment calculations.

Computational toxicology and in silico calculations can be a valuable tool in the context of standard approaches used in toxicity prediction, mechanistic rationale, and health risk assessment. Perin and colleagues used a similar approach in the assessment of organic compounds detected in surface water [73]. In the present work, and in the context of demonstrating the importance of in silico toxicity evaluation, using the Derek Nexus in silico tool for both acetaminophen and caffeine, an informative overview of the potentially toxic effects of those compounds was achieved, which can be partially contrasted and discussed with the concentration found in fish samples. Among the 62 endpoints investigated via the in silico tool, 54 did not result in any alarming effect at the selected reasoning level. Exact matches for acetaminophen were reported for two endpoints. Acetaminophen has been implicated (as a potential causative agent) in hepatotoxicity effects after long-term use and intentional dosing by ingestion (i.e., >4 g/day, as maximum dose advocated on the package) [74]. Even though they are not directly comparable (administered levels of pharmaceuticals in contrast to traces detected in fish commodity), the herein detected levels for both compounds are far below the concentrations reported; therefore, realistic consumption of such fish will possibly not lead to an uptake of such high concentrations. Overall, the in silico toxicity tool adds another evidence to the outcome corroborated in the HQ calculations.

## 5. Conclusions

A battery of analytical methods covering more than 280 chemicals exemplified by pesticides, pharmaceuticals, and macro and trace elements was applied to fish samples collected from Thermaikos Gulf, one of the major fishing grounds in Greece. The low prevalence of pesticides and pharmaceuticals was corroborated, with overall concentrations not exceeding 14 μg/Kg fish. The latter signifies that despite the intensive agricultural and industrial activity in the extended coastal area of Thermaikos Gulf, the chemical concentrations still remain low. As a general observation, we underline that the results from a human health risk assessment perspective are of particular importance due to the presence of some heavy metals, considering the potential threat that may pose to children after the long-term consumption of fish. However, in no case these results can be used for actual and reliable risk–benefit analysis of fish consumption for human health due to the very limited number of samples and lack of any systematic monitoring methodology. Future steps include the extended sampling of fish species, the incorporation of CRM negative control fish tissue samples that will strengthen the validation of the analytical methods and decrease analytical uncertainty, further extension of the analytical scope to pesticides and pharmaceuticals’ metabolites, the speciation analysis of As and Cr (both inorganic and organic forms), and the untargeted chemical analysis via high-resolution mass spectrometry systems to address the totality of pollutants in the specific marine environment.

## Figures and Tables

**Table 1:** Calculated ADD and HQ values for all the detected pesticides, pharmaceuticals, and toxic elements, considering the worst cases.

				ADD (μg/Kg/day) *		HQ **	
Elements	TDI (μg/Kg/day)	Concentration (μg/Kg)		Adults	Children	Adults	Children
Pb	2 [48]	38		0.03	0.14	0.02	0.07
As	0.3 [49]	125		0.10	0.47	0.33	1.56
Cr	3 [50]	796		0.64	2.97	0.21	0.99
Ni	13 [51]	1422		1.14	5.31	0.09	0.41
Hg	0.1 [52]	128		0.10	0.48	1.02	4.76
				**ADD (μg/Kg/day)**		**HQ**	
**Compounds**	**TDI** **(μg/Kg/day)**	**Concentration (μg/Kg fish)**	**Concentration (μg/Kg d.w.)**	**Adults**	**Children**	**Adults**	**Children**
Thiabendazole	100 [53]	13.7	68.5	5.48 × 10^−2^	2.56 × 10^−1^	5.48 × 10^−4^	2.56 × 10^−3^
Cypermethrin	5 [54]	8.7	43.5	3.48 × 10^−2^	1.62 × 10^−1^	6.96 × 10^−3^	3.25 × 10^−2^
Tricyclazole	50 [55]	0.9	4.5	3.60 × 10^−3^	1.68 × 10^−2^	7.20 × 10^−5^	3.36 × 10^−4^
Caffeine	2.5 [56]	3.8	19	1.52 × 10^−2^	7.09 × 10^−2^	6.08 × 10^−3^	2.84 × 10^−2^
Acetaminophen	300 [56]	1.5	7.5	6.00 × 10^−3^	2.80 × 10^−2^	2.00 × 10^−5^	9.33 × 10^−5^

* ADD: Average daily intake. ** HQ: Hazard quotient.

## Data Availability

All data are presented in the manuscript and its supplementary material.

## Supplementary Materials

The following supporting information can be downloaded at: https://www.mdpi.com/article/10.3390/jox14020041/s1, Table S1: Analytical scope (pesticides), LOQs and recoveries (at the LOQ) obtained in fish; Table S2: LODs, LOQs, and recoveries (including standard deviation, SD) obtained for pharmaceuticals in whole fish; Table S3: MRM conditions for antibiotics and pharmaceuticals in LC-ESI-MS/MS; Table S4: Concentrations of trace elements (mg/kg) in each individual fish sample; Table S5: Concentrations of macro elements (mg/kg) in each individual fish sample; Table S6: Quantification parameters of trace and macro elements; Table S7: Calculated HQ (adult, children) values for the most toxic elements; Figure S1: MRM chromatograms of *o,p’*-DDE traces (in proximity to the LOD) in seabass; Figure S2: MRM chromatograms of caffeine in: (a) blank fish sample, (b) in spiked fish sample at 5 μg/Kg fish, and (c) MRM chromatogram (quantitation) of ibuprofen traces (at LOD) in *D. labrax.*
