# Supplementary material for: Bioaccumulation of Organic and Inorganic Pollutants in Fish from Thermaikos Gulf: Preliminary Human Health Risk Assessment Assisted by a Computational Approach
Source: J Xenobiot. 2024 Jun 1;14(2):701–16. doi: 10.3390/jox14020041 (PMC11204809; doi:10.3390/jox14020041)
Supplement: Supplementary file 1 [file jox-14-00041-s001.zip › jox-2987628-supplementary.pdf]

SUPPLEMENTARY DATA to the paper entitled:

Bioaccumulation of Organic and Inorganic Pollutants in Fish from Thermaikos Gulf: Preliminary Human Health Risk Assessment Assisted by a Computational Approach

Konstantinos M. Kasiotis [1,*], Effrosyni Zafeiraki [1,*], Electra Manea-Karga [1], Demetrios Kouretas [2], Fotis Tekos [2],
Zoi Skaperda [2], Nikolaos Doumpas [3] and Kyriaki Machera [1]

[1] Laboratory of Pesticides' Toxicology, Department of Pesticides Control and Phytopharmacy, Benaki Phytopathological Institute, 8 Stefanou Delta Str., 14561 Athens, Greece; e.manea-karga@bpi.gr (E.M.-K.); k.machera@bpi.gr (K.M.)
[2] Department of Biochemistry and Biotechnology, University of Thessaly, Viopolis, Mezourlo, 41500 Larissa, Greece; dkouret@uth.gr (D.K.); fotis.tek@gmail.com (F.T.); skaperda@foodoxys.com (Z.S.)
[3] iSea, Environmental Organisation for the Preservation of the Aquatic Ecosystems, 54645 Thessaloniki, Greece; nikolaos.doumpas@isea.com.gr (N.D.)
* Correspondence: k.kasiotis@bpi.gr (K.M.K.); e.zafeiraki@bpi.gr (E.Z.); Tel.: +30-2108180357 (K.M.K. & E.Z.)

This document provides more detailed information to the main paper mentioned above.
The following information includes:

**Content**		**Page**
Table S1	Analytical scope (pesticides), LOQs and recoveries (at the LOQ) obtained in fish.	2
Table S2	LODs, LOQs, and recoveries (including standard deviation, SD) obtained for pharmaceuticals in whole fish	8
Table S3	MRM conditions for antibiotics and pharmaceuticals in LC-ESI-MS/MS.	9
Table S4	Concentrations of trace elements (μg/kg) in each individual fish sample.	11
Table S5	Concentrations of macro elements (mg/kg) in each individual fish sample.	12
Table S6	Quantification parameters of trace and macro elements.	13
Table S7	Calculated HQ (adult, children) values for the most toxic elements.	15
Figure S1	MRM chromatograms of *o,p′*-DDE traces (in proximity to the LOD) in seabass	16
Figure S2	MRM chromatograms of caffeine in: a) blank fish sample, b) in spiked fish sample at 5 μg/Kg fish, and c) MRM chromatogram (quantitation) of ibuprofen traces (at LOD) in *D. labrax*	16

Table S1: Analytical scope, LOQs and recoveries (at the LOQ) obtained in fish

No	Active substances	Quantification method	LOQ (μg/Kg fish)	Recovery±SD (n=3)	No	Active substances	Quantification method	LOQ (μg/Kg fish)	Recovery±SD (n=3)
**1**	Acephate	LC-ESI-MS/MS	1	75±11	**126**	Fluometuron	LC-ESI-MS/MS	5	69±8
**2**	Acetamiprid	LC-ESI-MS/MS	1	80±12	**127**	Fluopicolide	LC-ESI-MS/MS	5	75±6
**3**	Acrinathrin	GC-MS/MS	5	78±8	**128**	Fluopyram	LC-ESI-MS/MS	1	82±9
**4**	Alachlor	GC-MS/MS	1	75±7	**129**	Fluquinconazole	LC-ESI-MS/MS	1	75±5
**5**	Aldicarb	LC-ESI-MS/MS	5	80±8	**130**	Flusilazole	LC-ESI-MS/MS	1	70±9
**6**	Aldicarb sulfone	LC-ESI-MS/MS	1	76±7	**131**	Flutriafol	LC-ESI-MS/MS	5	78±11
**7**	Aldicarb sulfoxide	LC-ESI-MS/MS	1	80±8	**132**	Fluxapyroxad	LC-ESI-MS/MS	1	82±8
**8**	Aldrin	GC-MS/MS	1	80±13	**133**	Fosthiazate	LC-ESI-MS/MS	1	68±6
**9**	Ametryn	LC-ESI-MS/MS	5	83±14	**134**	Furathiocarb	LC-ESI-MS/MS	1	75±8
**10**	Amitraz	LC-ESI-MS/MS	5	85±8	**135**	Fuberidazole	LC-ESI-MS/MS	5	82±13
**11**	Atrazine	LC-ESI-MS/MS	5	76±10	**136**	Haloxyfop	LC-ESI-MS/MS	5	74±13
**12**	Avermectin B1a	LC-ESI-MS/MS	1	70±7	**137**	HCH-a	GC-MS/MS	1	77±7
**13**	Avermectin B1b	LC-ESI-MS/MS	5	71±8	**138**	HCH-b	GC-MS/MS	1	80±10
**14**	Azinphos ethyl	GC-MS/MS	5	80±10	**139**	Heptachlor	GC-MS/MS	1	79±7
**15**	Azinphos methyl	LC-ESI-MS/MS	5	76±4	**140**	Heptenophos	LC-ESI-MS/MS	5	84±12
**16**	Azoxystrobin	LC-ESI-MS/MS	5	86±11	**141**	Hexachlorobenzene	GC-MS/MS	1	73±6
**17**	Benalaxyl	LC-ESI-MS/MS	5	79±13	**142**	Hexaconazole	LC-ESI-MS/MS	1	82±15
**18**	Benfuracarb	LC-ESI-MS/MS	1	69±6	**143**	Hexythiazox	LC-ESI-MS/MS	5	70±12

**19**	Bifenthrin	GC-MS/MS	5	85±7	**144**	5-Hydroxy imidacloprid	LC-ESI-MS/MS	5	77±12
**20**	Biphenyl	GC-MS/MS	5	77±10	**145**	Imazalil	LC-ESI-MS/MS	1	90±12
**21**	Boscalid	LC-ESI-MS/MS and GC-MS/MS	5	81±5	**146**	Imidacloprid	LC-ESI-MS/MS	0.5	91±8
**22**	Bromacil	LC-ESI-MS/MS	5	78±10	**147**	Imazamethabenz-methyl	LC-ESI-MS/MS	5	73±7
**23**	Bromopropylate	GC-MS/MS	1	87±6	**148**	Indoxacarb	LC-ESI-MS/MS	1	77±12
**24**	Bromuconazole	LC-ESI-MS/MS	1	72±5	**149**	Iprodione	LC-ESI-MS/MS	5	71±9
**25**	Bupirimate	LC-ESI-MS/MS	5	78±8	**150**	Iprovalicarb	LC-ESI-MS/MS	1	79±15
**26**	Buprofezin	LC-ESI-MS/MS	5	81±11	**151**	Isophenphos methyl	GC-MS/MS	5	68±7
**27**	Caduzafos	LC-ESI-MS/MS	1	75±11	**152**	Isoprothiolane	LC-ESI-MS/MS	5	78±9
**28**	Carbaryl	LC-ESI-MS/MS	5	79±4	**153**	Isoproturon	LC-ESI-MS/MS	5	72±3
**29**	Carbendazim	LC-ESI-MS/MS	5	92±8	**154**	Lindane (HCH gamma)	GC-MS/MS	1	72±3
**30**	Carbofuran	LC-ESI-MS/MS	1	80±9	**155**	Linuron	LC-ESI-MS/MS	1	92±6
**31**	Carbofuran 3 hydroxy	LC-ESI-MS/MS	5	77±10	**156**	Malaoxon	LC-ESI-MS/MS	1	79±10
**32**	Carbosulfan	LC-ESI-MS/MS	5	72±8	**157**	Malathion	LC-ESI-MS/MS	5	91±15
**33**	Chlorantranilipr ole	LC-ESI-MS/MS	5	75±5	**158**	Mepanipyrim	LC-ESI-MS/MS	5	72±10
**34**	Chlordane a	GC-MS/MS	1	84±11	**159**	Metalaxyl M	LC-ESI-MS/MS	1	89±7
**35**	Chlorfenvinfos	GC-MS/MS	1	81±11	**160**	Metamitron	LC-ESI-MS/MS	1	89±10
**36**	Chloridazon	LC-ESI-MS/MS	1	73±5	**161**	Metazachlor	LC-ESI-MS/MS	1	69±8
**37**	Chlorobenzilate	GC-MS/MS	1	74±7	**162**	Metconazole	LC-ESI-MS/MS	5	81±7
**38**	Chlorobromuron	LC-ESI-MS/MS	5	81±7	**163**	Methamidophos	LC-ESI-MS/MS	5	89±7
**39**	Chloroxuron	LC-ESI-MS/MS	5	84±8	**164**	Methidathion	GC-MS/MS	5	75±8

**40**	Chlorpropham	LC-ESI-MS/MS	1	76±6	**165**	Methiocarb	LC-ESI-MS/MS	1	76±7
**41**	Chlorpyriphos ethyl	LC-ESI-MS/MS and GC-MS/MS	1	79±7	**166**	Methiocarb sulfone	LC-ESI-MS/MS	1	81±11
**42**	Chlorpyrifos oxon	LC-ESI-MS/MS	1	80±12	**167**	Methiocarb sulfoxide	LC-ESI-MS/MS	1	77±13
**43**	Chlorpyriphos methyl	LC-ESI-MS/MS	5	77±4	**168**	Methomyl	LC-ESI-MS/MS	1	84±15
**44**	Chlorthal dimethyl	GC-MS/MS	5	85±8	**169**	Methoxyfenozide	LC-ESI-MS/MS	5	73±13
**45**	Clofentezine	LC-ESI-MS/MS	5	78±11	**170**	Metobromuron	LC-ESI-MS/MS	1	75±5
**46**	Clopyralid	LC-ESI-MS/MS	5	80±9	**171**	Metolachlor	LC-ESI-MS/MS	5	78±16
**47**	Clothianidin	LC-ESI-MS/MS	5	85±12	**172**	Metraferone	LC-ESI-MS/MS	5	75±7
**48**	Coumaphos	LC-ESI-MS/MS and GC-MS/MS	1	81±6	**173**	Mevinphos cis/trans (cis+trans)	LC-ESI-MS/MS	1	82±16
**49**	Coumaphos oxon	LC-ESI-MS/MS	1	85±10	**174**	Monocrotophos	LC-ESI-MS/MS	1	77±11
**50**	Cyfluthrin	GC-MS/MS	1	80±9	**175**	Monolinuron	LC-ESI-MS/MS	5	68±11
**51**	λ-Cyhalothrin	GC-MS/MS	1	85±7	**176**	Myclobutanil	LC-ESI-MS/MS	1	70±5
**52**	Cypermethrin	GC-MS/MS	1	79±12	**177**	Napropamide	LC-ESI-MS/MS	5	73±7
**53**	Cyproconazole	LC-ESI-MS/MS	1	78±8	**178**	Nitenpyram	LC-ESI-MS/MS	1	76±11
**54**	Cyprodinil	LC-ESI-MS/MS	5	81±9	**179**	Omethoate	LC-ESI-MS/MS	1	82±6
**55**	DDT pp	GC-MS/MS	0.5	80±8	**180**	Oxadixyl	LC-ESI-MS/MS	5	72±5
**56**	DDE o,p	GC-MS/MS	0.5	81±13	**181**	Oxamyl	LC-ESI-MS/MS	5	80±14
**57**	DDE pp	GC-MS/MS	0.5	84±7	**182**	Oxyfluorfen	LC-ESI-MS/MS	5	79±7
**58**	DDD pp	GC-MS/MS	0.5	84±7	**183**	Paclobutazole	LC-ESI-MS/MS	5	80±5
**59**	Deltamethrin	GC-MS/MS	1	75±5	**184**	Paraoxon methyl	GC-MS/MS	5	70±8

**60**	Demeton S methyl	LC-ESI-MS/MS	1	81±13	**185**	parathion ethyl	LC-ESI-MS/MS	5	80±9
**61**	Demeton S methyl sulfone	LC-ESI-MS/MS	1	84±10	**186**	Parathion methyl	LC-ESI-MS/MS	5	75±10
**62**	Demeton S methyl sulfoxide	LC-ESI-MS/MS	1	87±9	**187**	Penconazole	LC-ESI-MS/MS	1	73±12
**63**	Diazinon	LC-ESI-MS/MS	1	83±5	**188**	Pencycuron	LC-ESI-MS/MS	5	85±15
**64**	Dichlofluanid	LC-ESI-MS/MS	1	81±13	**189**	Pendimethalin	LC-ESI-MS/MS	5	77±16
**65**	Dichlorvos	LC-ESI-MS/MS	5	74±8	**190**	Permethrin	GC-MS/MS	1	76±8
**66**	Dicofol op	GC-MS/MS	5	84±16	**191**	Phorate	GC-MS/MS	5	69±9
**67**	Dicofol pp	GC-MS/MS	5	81±14	**192**	Phosalone	LC-ESI-MS/MS	5	85±17
**68**	Dicrotofos (dicrotophos)	LC-ESI-MS/MS	1	79±9	**193**	Phosmet	LC-ESI-MS/MS	1	86±7
**69**	Dieldrin	GC-MS/MS	1	85±13	**194**	Phosmet oxon	LC-ESI-MS/MS	5	78±10
**70**	Diethofencarb	LC-ESI-MS/MS	1	82±6	**195**	Phoxim	LC-ESI-MS/MS	1	74±12
**71**	Difenoconazole	LC-ESI-MS/MS	5	73±8	**196**	Picoxystrobin	LC-ESI-MS/MS	5	90±12
**72**	Diflubenzuron	LC-ESI-MS/MS	5	88±8	**197**	Pirimicarb	LC-ESI-MS/MS	5	76±7
**73**	Dimethoate	LC-ESI-MS/MS	1	90±11	**198**	Pirimicarb desmethyl	LC-ESI-MS/MS	1	81±10
**74**	Dimethomorph	LC-ESI-MS/MS	1	81±7	**199**	Pirimiphos methyl	LC-ESI-MS/MS	1	79±6
**75**	Diniconazole	LC-ESI-MS/MS	1	74±4	**200**	Procloraz	LC-ESI-MS/MS	5	69±4
**76**	Diphenylamine	LC-ESI-MS/MS	5	80±17	**201**	Prometryn	LC-ESI-MS/MS	5	83±9
**77**	Disulfoton	LC-ESI-MS/MS	1	82±11	**202**	Propamocarb	LC-ESI-MS/MS	1	102±20
**78**	Disulfoton sulfoxide	LC-ESI-MS/MS	5	85±10	**203**	Propargite	LC-ESI-MS/MS	5	107±17
**79**	Diuron	LC-ESI-MS/MS	1	78±10	**204**	Propoxur	LC-ESI-MS/MS	5	85±7
**80**	DMF	LC-ESI-MS/MS	5	79±5	**205**	propyconazole	LC-ESI-MS/MS	5	74±7

**81**	DMPF	LC-ESI-MS/MS	5	85±10	**206**	Propyzamide	GC-MS/MS	5	70±10
**82**	Dodemorph	LC-ESI-MS/MS	1	91±13	**207**	Prothioconazole desthio	LC-ESI-MS/MS	5	81±8
**83**	Emamectin B1a	LC-ESI-MS/MS	5	75±14	**208**	Prothiofos	LC-ESI-MS/MS	1	79±12
**84**	Emamectin B1b	LC-ESI-MS/MS	5	76±9	**209**	Pymethrozine	LC-ESI-MS/MS	5	81±14
**85**	Endosulfan a	GC-MS/MS	5	85±13	**210**	Pyraclostrobin	LC-ESI-MS/MS	1	103±14
**86**	Endosulfan b	GC-MS/MS	1	82±9	**211**	Pyrazophos	LC-ESI-MS/MS	1	72±5
**87**	Endosulfan sulfate	GC-MS/MS	1	90±10	**212**	Pyridaben	LC-ESI-MS/MS	5	83±7
**88**	Endrin	GC-MS/MS	1	78±12	**213**	Pyrifenox	LC-ESI-MS/MS	5	101±18
**89**	Epoxiconazole	LC-ESI-MS/MS	1	85±15	**214**	Pyrimethanil	LC-ESI-MS/MS	5	79±8
**90**	Esfenvalerate	GC-MS/MS	1	83±20	**215**	Pyriproxyfen	LC-ESI-MS/MS	1	102±12
**91**	Ethion	GC-MS/MS	5	80±9	**216**	Quinalphos	LC-ESI-MS/MS	5	69±10
**92**	Ethirimol	LC-ESI-MS/MS	1	78±5	**217**	Quinoxyfen	LC-ESI-MS/MS	5	80±11
**93**	Ethofumesate	LC-ESI-MS/MS	5	77±13	**218**	Quintozene	GC-MS/MS	5	81±9
**94**	Ethoprophos	LC-ESI-MS/MS	1	74±8	**219**	Quizalofop	LC-ESI-MS/MS	5	76±11
**95**	Ethoxyquin	LC-ESI-MS/MS	5	76±6	**220**	Spinosad (A)	LC-ESI-MS/MS	1	91±9
**96**	Etofenprox	LC-ESI-MS/MS	5	85±10	**221**	Spinosad (D)	LC-ESI-MS/MS	1	90±12
**97**	Etoxazole	LC-ESI-MS/MS	5	78±8	**222**	Spirodiclofen	LC-ESI-MS/MS	1	77±6
**98**	Famoxadone	LC-ESI-MS/MS	5	76±13	**223**	Spiromesifen	GC-MS/MS	5	72±4
**99**	Fenamidone	LC-ESI-MS/MS	5	82±12	**224**	Spiroxamine (I + II)	LC-ESI-MS/MS	1	81±6
**100**	Fenamiphos	LC-ESI-MS/MS	1	78±12	**225**	Sulfoxaflor	LC-ESI-MS/MS	5	79±15
**101**	Fenamiphos sulfone	LC-ESI-MS/MS	1	83±12	**226**	Tau-fluvalinate	GC-MS/MS	1	78±8
**102**	Fenamiphos sulfoxide	LC-ESI-MS/MS	5	86±8	**227**	Tebuconazole	LC-ESI-MS/MS	5	74±10

**103**	Fenarimol	LC-ESI-MS/MS	5	78±18	**228**	Tebufenozide	LC-ESI-MS/MS	5	78±8
**104**	Fenazaquin	LC-ESI-MS/MS	5	81±12	**229**	Tebufenpyrad	LC-ESI-MS/MS	1	81±14
**105**	Fenbuconazole	LC-ESI-MS/MS	5	73±13	**230**	Terbufos	LC-ESI-MS/MS	1	80±8
**106**	Fenhexamide	LC-ESI-MS/MS	1	78±8	**231**	Terbuthylazine	LC-ESI-MS/MS	1	90±11
**107**	Fenitrothion	LC-ESI-MS/MS	1	70±13	**232**	Terbutryn	LC-ESI-MS/MS	5	67±12
**108**	Fenoxycarb	LC-ESI-MS/MS	5	78±8	**233**	Tetrachlorvinphos	LC-ESI-MS/MS	5	75±8
**109**	Fenpropathrin	GC-MS/MS	1	72±16	**234**	Tetraconazole	LC-ESI-MS/MS	5	83±12
**110**	Fenpropidin	LC-ESI-MS/MS	1	82±10	**235**	Tetradifon	GC-MS/MS	5	100±8
**111**	Fenpropymorph	LC-ESI-MS/MS	5	78±19	**236**	Thiabendazole	LC-ESI-MS/MS	1	71±6
**112**	Fenpyroximate	LC-ESI-MS/MS	5	72±5	**237**	Thiacloprid	LC-ESI-MS/MS	1	93±10
**113**	Fenthion	GC-MS/MS	5	85±18	**238**	Thiamethoxam	LC-ESI-MS/MS	1	88±9
**114**	Fenthion oxon (fenoxon)	LC-ESI-MS/MS	1	85±10	**239**	Thiobencarb	LC-ESI-MS/MS	1	90±10
**115**	Fenthion oxon sulfone	LC-ESI-MS/MS	1	75±8	**240**	Thiodicarb	LC-ESI-MS/MS	1	90±7
**116**	Fenthion oxon sullfoxide	LC-ESI-MS/MS	1	82±7	**241**	Thiophanate methyl	LC-ESI-MS/MS	1	92±18
**117**	Fenthion sulfone	LC-ESI-MS/MS	1	85±11	**242**	Tolclofos methyl	GC-MS/MS	1	85±17
**118**	Fenthion sulfoxide	LC-ESI-MS/MS	1	76±13	**243**	Triadimefon	LC-ESI-MS/MS	1	72±6
**119**	Fenvalerate	GC-MS/MS	5	81±11	**244**	Triadimenol	LC-ESI-MS/MS	1	68±4
**120**	Flonicamid	LC-ESI-MS/MS	5	90±10	**245**	Triazophos	LC-ESI-MS/MS	1	73±12
**121**	Fluazifop	LC-ESI-MS/MS	5	91±10	**246**	Tricyclazole	LC-ESI-MS/MS	0.5	74±4
**122**	Fluazifop-P-butyl	LC-ESI-MS/MS	5	82±7	**247**	Trifloxystrobin	LC-ESI-MS/MS	1	110±14
**123**	Fludioxonil	LC-ESI-MS/MS	5	85±10	**248**	Trifluralin	GC-MS/MS	5	75±14
**124**	Flufenoxuron	LC-ESI-MS/MS	5	71±5	**249**	Vinclozolin	GC-MS/MS	5	76±3

**125**	Flufenacet	LC-ESI-MS/MS	5	75±8	**250**	Zoxamide	LC-ESI-MS/MS	5	82±5

Table S2. LODs, LOQs, and recoveries (including standard deviation, SD) obtained for pharmaceuticals in whole fish

			**Recoveries (%)**					
**Compounds**	**LOD (µg/Kg fish)**	**LOQ (µg/Kg fish)**	**LOQ**	**SD (n=3)**	**5 × LOQ**	**SD (n=3)**	**50 × LOQ**	**SD (n=3)**
**Acetaminophen**	0.38	1.15	89	5	90	8	104	7
**Amoxicillin**	1.25	3.76	81	10	82	11	87	8
**Atorvastatin**	1.35	4.05	69	5	74	10	75	10
**Azithromycin**	2.75	8.25	66	6	65	9	68	11
**Caffeine**	0.68	2.05	64	5	68	7	71	8
**Cefuroxim**	0.73	2.18	64	4	71	13	74	12
**Clarithromycin**	1.02	3.05	67	5	72	4	71	7
**Ibuprofen**	0.82	2.46	78	12	83	8	84	9
**Ramipril**	0.65	1.95	75	8	76	12	79	7
**Venlafaxine**	1.10	3.30	77	7	78	10	82	9

Table S3. MRM conditions for antibiotics and pharmaceuticals in LC-ESI-MS/MS.

**Retention time (min)**	**Compound Name**	**Precursor Ion**	**Product Ion***	**Dwell time**	**Fragmentor Voltage**	**Collision Energy**	**Polarity**
14.25	Atorvastatin	559	250	50	50	36	Positive
		559	**440**	50	50	18	Positive
12.27	Venlafaxine	278.1	**57.8**	50	100	18	Positive
		278.1	260.3	50	100	12	Positive
13.38	Ramipril	417.2	117	50	132	40	Positive
		417.2	**234.1**	50	132	16	Positive
14.37	Ibuprofen**	205.2	161.1	15	100	20	Negative
13.69	Clarithromycin	748.5	590.3	50	10	12	Positive
		748.5	**158.1**	50	10	24	Positive
		748.5	116.1	50	10	40	Positive
10.74	Caffeine	195.1	110.1	50	132	20	Positive
		195.1	**138**	50	132	16	Positive
13.68	Azithromycin	749.5	**158.1**	50	10	32	Positive
		749.5	116.1	50	10	44	Positive
16.69	Amoxicillin	366.2	114	50	68	22	Positive

		349	**255**	15	70	18	Positive
		349	107	15	70	36	Positive
4.26	Acetaminophen	152.1	**110.1**	50	107	12	Positive
		152.1	65	50	107	32	Positive
17.04	Cefuroxime	423.2	206.7	25	70	15	Negative
		423.2	**317.8**	25	70	10	Negative

*in bold the quantitation ion, **for ibuprofen two MRM transitions were obtained in positive ionization as well, but were less abundant than the one in negative

Table S4: Concentrations of trace elements (μg/Kg) in each individual fish sample.

Sample code	Species	Common name	Al	Ti	V	Cr	Mn	Fe	Co	Ni	Cu	Zn	As	Se	Sr	Mo	Ag	Cd	Sn	Sb	Ba	Tl	Pb	Hg	U
1	*Dicentrarchus labrax (D.labrax)*	Sea bass	1503	428	26	796	917	19853	26	1422	873	10166	45	279	17341	41	<LOQ	<LOQ	23	3,7	<LOQ	<LOQ	22	75	<LOQ
2	*Dicentrarchus labrax (D.labrax)*	Sea bass	1020	226	26	145	588	4830	<LOQ	756	433	8352	36	473	19663	<LOQ	<LOQ	<LOQ	6,8	4,4	<LOQ	<LOQ	31	101	<LOQ
3	*Dicentrarchus labrax (D.labrax)*	Sea bass	1343	285	34	259	543	9731	<LOQ	123	638	11004	74	201	24047	<LOQ	<LOQ	<LOQ	3,8	2,2	<LOQ	<LOQ	38	128	<LOQ
4	*Dicentrarchus labrax (D.labrax)*	Sea bass	2744	524	<LOQ	190	458	5831	<LOQ	100	<LOQ	7028	20	173	9857	<LOQ	<LOQ	<LOQ	4,0	1,9	<LOQ	<LOQ	22	70	<LOQ
5	*Dicentrarchus labrax (D.labrax)*	Sea bass	3006	333	<LOQ	747	533	7387	<LOQ	43	470	7349	56	216	15272	<LOQ	5.4	<LOQ	3,8	3,8	<LOQ	<LOQ	25	84	<LOQ
6	*Solea Solea (S.solea)*	Sole	1964	240	27	59	4546	5862	<LOQ	126	<LOQ	4113	75	200	16925	<LOQ	<LOQ	<LOQ	1,7	1,6	<LOQ	<LOQ	21	88	<LOQ
7	*Solea Solea (S.solea)*	Sole	2205	274	<LOQ	146	3324	4777	<LOQ	148	<LOQ	5370	103	143	15262	<LOQ	<LOQ	<LOQ	2,2	1,7	<LOQ	<LOQ	26	78	<LOQ
8	*Solea Solea (S.solea)*	Sole	4019	459	<LOQ	243	3266	5804	<LOQ	83	<LOQ	4511	126	155	22740	<LOQ	<LOQ	<LOQ	4,9	4,6	<LOQ	<LOQ	32	104	<LOQ
9	*Solea Solea (S.solea)*	Sole	1866	254	<LOQ	154,	4313	2648	<LOQ	95	<LOQ	3640	80	159	13162	<LOQ	<LOQ	<LOQ	5,3	3,1	<LOQ	<LOQ	24	100	<LOQ
10	*Solea Solea (S.solea)*	Sole	3299	285	<LOQ	162	3477	4750	<LOQ	43	<LOQ	5058	75	211	16314	<LOQ	<LOQ	<LOQ	6,8	4,5	<LOQ	<LOQ	29	86	<LOQ

Table S5: Concentrations of macro elements (mg/Kg) in each individual fish sample.

Sample code	**Species**	**Common name**	**Mg**	**P**	**K**	**Ca**
**1**	*Dicentrarchus labrax (D.labrax)*	Sea bass	280	3100	3431	4256
**2**	*Dicentrarchus labrax (D.labrax)*	Sea bass	353	3655	3674	5405
**3**	*Dicentrarchus labrax (D.labrax)*	Sea bass	337	3681	3975	5046
**4**	*Dicentrarchus labrax (D.labrax)*	Sea bass	322	2391	3526	2492
**5**	*Dicentrarchus labrax (D.labrax)*	Sea bass	303	3084	3896	4317
**6**	*Solea Solea (S.solea)*	Sole	318	2917	4056	3505
**7**	*Solea Solea (S.solea)*	Sole	337	3155	4279	4239
**8**	*Solea Solea (S.solea)*	Sole	351	3332	3570	4983
**9**	*Solea Solea (S.solea)*	Sole	287	2503	3589	2888
**10**	*Solea Solea (S.solea)*	Sole	351	3024	4328	3537

Table S6: Quantification parameters of trace and macro elements.

Element	Symbol	Monitored isotopes	Reporting isotope	Interference correction	ISTD	Analysis mode	LOQ (mg/Kg)
Magnesium	*Mg*	*24*	*24*		^{45}Sc	KED	100
Aluminium	*Al*	*27*	*27*		^{45}Sc	KED	0.25
Phosphorus	*P*	*31*	*31*		^{45}Sc	KED	1
Potassium	*K*	*39*	*39*		^{45}Sc	KED	1
Calcium	*Ca*	*43. 44*	*44*		^{45}Sc	KED	100
Titanium	*Ti*	*47.49*	*47*		^{45}Sc	KED	0.025
Vanadium	*V*	*51*	*51*		^{45}Sc	KED	0.025
Chromium	*Cr*	*52. 53*	*52*		^{72}Ge	KED	0.025
Manganese	*Mn*	*55*	*55*		^{72}Ge	KED	0.1
Iron	*Fe*	*56. 57*	*56*		^{72}Ge	KED	0.25
Cobalt	*Co*	*59*	*59*		^{72}Ge	KED	0.025
Nickel	*Ni*	*60*	*60*		^{72}Ge	KED	0.025
Copper	*Cu*	*63. 65*	*63*		^{72}Ge	KED	0.25
Zinc	*Zn*	*64. 66. 67*	*66*	*64Zn:-0.0348659*60Ni (KED)*	^{72}Ge	KED	0.25
Arsenic	*As*	*75*	*75*		^{72}Ge	KED	0.01
Selenium	*Se*	*77. 78*	*78*	*78Se: -0304348*83Kr (KED)*	^{72}Ge	KED	0.01* 10^{-3}
Strontium	*Sr*	*86. 88*	*88*	*86Sr: -1.50435*83Kr (KED)*	^{89}Y	KED	0.5
Molybdenum	*Mo*	*95*	*95*		^{89}Y	KED	0.025

Silver	*Ag*	*107. 109*	*107*		^{115}In	KED	0.01
Cadmium	*Cd*	*111. 114*	*111*	*114Cd: -0.0268373*118Sn (KED)*	^{115}In	KED	0.01
Tin	*Sn*	*118*	*118*		^{115}In	KED	0.001
Antimony	*Sb*	*121*	*121*		^{159}Tb	KED	0.001
Barium	*Ba*	*137*	*137*		^{159}Tb	KED	0.25
Thallium	*Tl*	*203. 205*	*205*		^{191}Ir	KED	0.01
Lead	*Pb*	*206. 207. 208*	*208*	*208Pb:1*207Pb (KED) + 1*206Pb (KED)*	^{191}Ir	KED	0.01
Mercury	*Hg*	*200. 201. 202*	*202*		^{191}Ir	KED	0.002
Uranium	*U*	*238*	*238*		^{191}Ir	KED	0.01
**Internal standards**	**Symbol**	**Monitored isotopes**	**Reporting isotope**	**Interference correction**			
	Li	*6*	*6*				
	Sc	*45*	*45*				
	Ge	*72.73*	*73*				
	Y	*89*	*89*				
	In	*115*	*115*	*-0.0148637*118Sn (KED)*			
	Tb	*159*	*159*				
	Ir	*191.193*	*191*				

Table S7: Calculated HQ (adult, children) values for the most toxic elements.

	Pb			As			Cr			Ni				Hg	
Sample code	C (μg/Kg)	HQ		C (μg/Kg)	HQ		C (μg/Kg)	HQ		C (μg/Kg)	HQ		C (μg/Kg)	HQ	
		Adults	Children		Adults	Children		Adults	Children		Adults	Children		Adults	Children
1	22	0.01	0.04	45	0.12	0.56	796	0.21	0.99	1422	0.09	0.41	75	0.60	2.79
2	31	0.01	0.06	36	0.10	0.45	145	0.04	0.18	756	0.05	0.22	101	0.81	3.76
3	38	0.02	0.07	74	0.20	0.92	259	0.07	0.32	123	0.01	0.04	128	1.02	4.76
4	22	0.01	0.04	20	0.05	0.25	190	0.05	0.24	100	0.01	0.03	70	0.56	2.61
5	25	0.01	0.05	56	0.15	0.70	747	0.20	0.93	458	0.03	0.13	84	0.67	3.15
6	21	0.01	0.04	75	0.20	0.93	59	0.02	0.07	43	0.00	0.01	88	0.71	3.30
7	26	0.01	0.05	103	0.27	1.28	146	0.04	0.18	126	0.01	0.04	78	0.62	2.91
8	32	0.01	0.06	125	0.33	1.56	243	0.06	0.30	148	0.01	0.04	104	0.83	3.88
9	24	0.01	0.04	80	0.21	0.99	154	0.04	0.19	83	0.01	0.02	100	0.80	3.75
10	29	0.01	0.05	75	0.20	0.94	162	0.04	0.20	95	0.01	0.03	86	0.69	3.21

Figure S1: MRM chromatograms of *o,p'*-DDE traces (in proximity to the LOD) in seabass

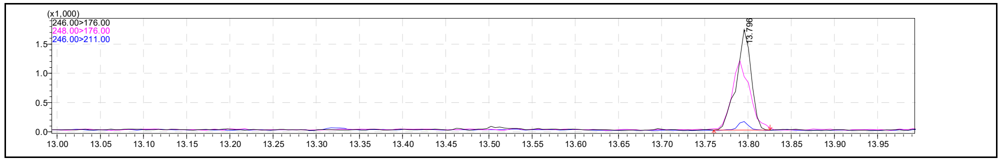

Figure S2: MRM chromatograms of caffeine in: a) blank fish sample, b) in spiked fish sample at 5 μg/Kg fish, and c) MRM chromatogram (quantitation) of ibuprofen traces (at LOD) in *D. labrax*

a

b

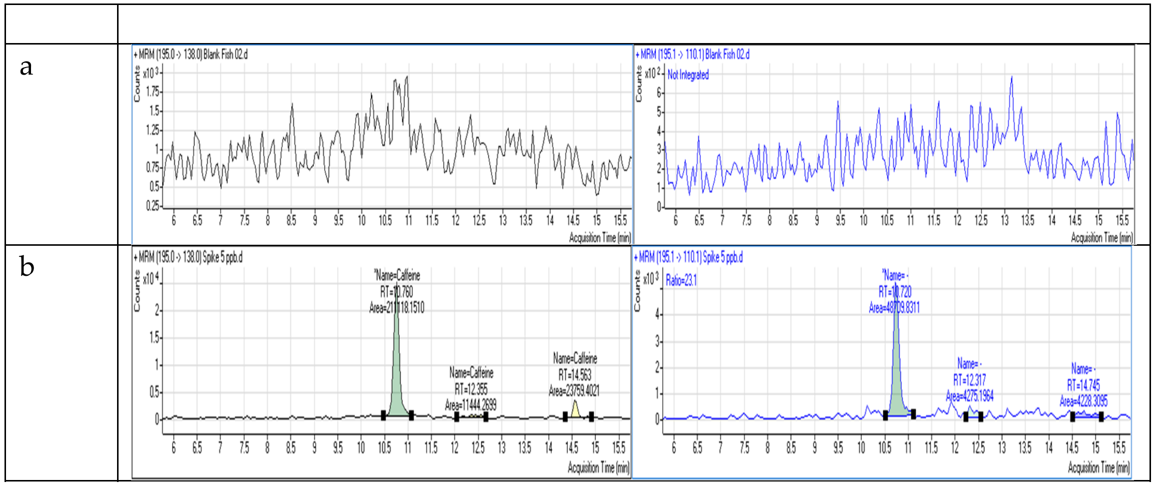

c	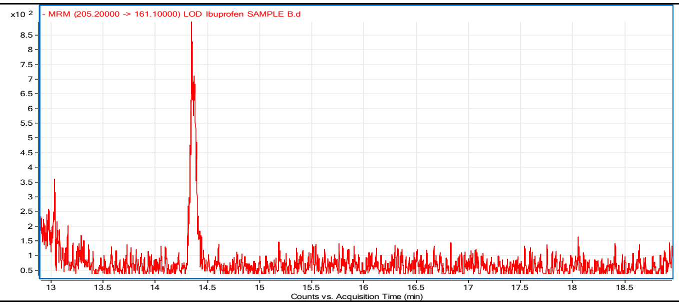x10 2<br>- MRM (205.20000 -> 161.10000) LOD Ibuprofen SAMPLE B.d<br>Counts vs. Acquisition Time (min)
